# Relationship between vertical jump tests and ice skating performance in junior Polish ice hockey players

**DOI:** 10.5114/biolsport.2023.112972

**Published:** 2022-03-16

**Authors:** Subir Gupta, Jakub Baron, Anna Bieniec, Andrzej Swinarew, Arkadiusz Stanula

**Affiliations:** 1 Faculty of Medical Sciences, University of West Indies, Cave Hill, Barbados; 2 Institute of Sport Sciences, Department of Exercise and Sport Performance, Jerzy Kukuczka Academy of Physical Education, Mikołowska 72A, 40-065 Katowice, Poland; 3 Faculty of Science and Technology, University of Silesia in Katowice, 75 Pułku Piechoty 1A, 41-500 Chorzów, Poland

**Keywords:** Countermovement jump, Squat jump, Depth drop jump, Skating multistage aerobic test, Repeated sprint ability, Maximum skating speed

## Abstract

The aim of this study was to determine the relationships between vertical jumps (VJ) and various on-ice skating performances of junior ice hockey players (n = 19). The three modes of VJ or off-ice measures were countermovement jump with arm swing (CMJ), squat jump (SJ) and depth drop jump (DDJ). The on-ice skating performance was measured by the skating multistage aerobic test (SMAT), forward and backward acceleration test, top speed test, and repeated sprint ability (RSA) test. The relationships between the variables were quantified using Pearson’s product-moment correlation. DDJ showed a significant positive correlation with forward average skating speed (FASS) (r = 0.62) and strong correlations with backward average skating speed (BASS) (r = 0.81), and maximum skating speed (MSS) (r = 0.71). SJ was found to be strongly correlated with BASS (r = 0.82) and MSS (r = 0.76), whereas the only on-ice performance that significantly correlated with CMJ was BASS (r = 0.68). All three modes of VJ were inversely and non-significantly correlated with performance decrement index and fatigue index, as determined by the RSA test. SMAT was not significantly correlated with either VJ or RSA. Correlations between all three modes of VJ tests were significant. Therefore, this study concludes that: (1) DDJ can be used as a predictor of all the ice skating speed tests, whereas SJ can predict BASS and MSS. CMJ, on the other hand, can predict the performance of only BASS. (2) RSA performance cannot be predicted from CMJ, SJ, or DDJ tests, and (3) neither any of the VJ nor RSA can predict skating endurance of junior ice hockey players.

## INTRODUCTION

Intermittent high intensity bouts of skating, rapid changes in direction and velocity, individual skills, tactics, and motivation are some important requirements of playing ice hockey. On-ice performance is a primary requirement for an ice hockey player and thus relevant on-ice assessment is an important component of the training programme for them. However, many of the suggested sports-specific tests of ice hockey are off-ice, and are performed in well-equipped laboratories or any suitable facility. Agility in on-ice performance is also related to off-ice conditioning. Commonly performed off-ice tests for ice hockey players are the Wingate test, various jump tests, repeated sprint ability (RSA) tests, and sprint tests or time trial run [1–6]. The validity of some of the off-ice tests has been questioned for quantifying aerobic or anaerobic power related to ice hockey [7, 8]. Off-ice tests mostly focus on both anaerobic endurance and aerobic capacities of the players, although the earlier one is predominant.

Studies confirmed a significant relationship between running speed and on-ice sprinting ability in young male ice hockey players [2, 9]. Jump tests are widely used to assess fitness of ice hockey players, and some of the jump tests are correlated with the performance of on-ice measurement [3, 4]. The jump test is used for measuring leg power and has repeatedly been shown to be related to skating abilities and ability to perform a wide range of simple and complex activities including throwing, sprinting, and change of direction [2, 4, 10]. Vertical jump is a part of the NHL combined test and very often is used not only as one of the gold standards, but as a simple off-ice test that can distinguish ice hockey potential [11, 12]. Vertical jumping is often considered as a basic movement skill, involves multi-joint movement and requires a higher level of motor coordination [2, 4]. The commonly used VJ test protocol varies widely. Countermovement jump with arm swing (CMJ), squat jump (SJ) and depth drop jump (DDJ) are the three major variations of VJ that were used in this study. Skating speed is one of the most desirable elements in ice hockey routinely assessed. Due to biomechanical similarities in movement patterns, strength and conditioning specialists recommend jumps and back squat exercise to improve ice skating performance [13].

Several previous studies focused on prediction of on-ice performance from off-ice tests [1, 2, 4, 8, 9, 13]. However, research focused on the relationship between various VJ tests and a wide range of on-ice performance, especially in junior ice hockey players, is scanty. Therefore, the primary purpose of this investigation was to determine whether any significant correlations exist between the (1) VJ tests and on-ice field tests, and (2) RSA and skating endurance (or SMAT) in junior Polish ice hockey players. It is hypothesized that DDJ is the single best off-ice VJ test in this study that predicts onice performance such as FASS, BASS, and MSS. Repeated sprint ability and SMAT, on the other hand, are not related to any of the VJ test performances.

## MATERIALS AND METHODS

### Subjects

Twenty-five outfield junior ice hockey players from the club UKS Zagłębie Sosnowiec of southern Poland participated in this study. Six players were excluded from the study after they failed to complete testing sessions. Of the final participants, 10 were defenders and 9 were forwards. Age, height, weight, and body mass index (BMI) of the players were 18.7 ± 2.2 years, 179.2 ± 11.6 cm, 77.8 ± 10.2 kg and 24.3 ± 1.9, respectively. The team was participating in the youth hockey league (MHL) and Central Junior League (CLJ) of Poland. The participants were in the middle of their competitive period, participating in 5–6 training sessions per week that comprised technical, tactical, fitness and strength training. The experimental protocol and potential risks involved in the study were described to all the participants and they were allowed to withdraw from any stage of the study without providing any reason. Participants, or their parents (in the case of < 18-year-old participants), gave voluntary written informed consent to participate in the study. The study was approved by the Ethics Committee of the Jerzy Kukuczka Academy of Physical Education in Katowice (No. 8/2018).

### Study design

This study was conducted over 4 days and on all the occasions the players were tested under similar experimental conditions and at the same time of day (6 to 9 p.m.). None of the volunteers ate food in the last 3 hours before testing, but water was allowed to drink. Participants were instructed to maintain normal dietary and fluid intake and to abstain from training and any other heavy physical activity for 24 hours before testing. Intake of coffee or any caffeine-containing beverages in the last 12 hours before the experiment was not allowed.

The whole study was conducted in two major stages – (1) off-ice VJ tests and (2) on-ice tests. The off-ice VJ tests conducted were CMJ, SJ, and DDJ. All off-ice VJ tests were conducted in the Laboratory of Analysis in Sports of the Jerzy Kukuczka Academy of Physical Education in Katowice (Poland). The four on-ice tests were – (i) skating multistage aerobic Test (SMAT or beep test), (ii) forward and backward acceleration test, (iii) top speed test, and (iv) repeated sprint ability (RSA) test. All the on-ice tests were conducted on the ice rink of Stadion Zimowy in Sosnowiec, Poland. All the on-ice and jump tests were explained and supervised by the same strength and conditioning specialist of the team. All the on-ice performances were tested wearing full ice hockey equipment with an ice hockey stick in their preferred hand.

### Vertical jump (VJ) tests

The subjects performed a warm-up, as instructed by the coach, just before performing the actual jump tests. All the jump tests were performed on the OptoJump (Microgate, Bolzano, Italy), an optical data acquisition system. The OptoJump is able to measure all air- and ground-related times with precision to 1/1000 of a second [14]. All subjects completed 3 trials for each jump test and the highest jump was recorded. Between two trials, a 1-min rest period was allowed. All the VJ tests were performed without any familiarization session. In order to control for the testing order effect, the order in which the players performed jump tests was randomized [15, 16].

### Countermovement jump with arm swing (CMJ)

The subject stood comfortably, bent his knees, hips and ankles, with his hands at his sides. Thereafter he was instructed to swing his arms forward when he jumped vertically. The countermovement started when the legs were bent down to 90°. A pre-jump or step was not allowed. Bending the knees before landing or kicking the legs forward by flexing at the hip was not allowed and the jump was repeated [17, 18].

### Squat jump (SJ)

The subject was instructed to stand with his hands on his hips and to bend at the knees, hips, and ankles. After he became motionless, the tester instructed him to jump. The player jumped as high as possible keeping his hands on his hips and his legs straight [19].

### Depth drop jump (DDJ)

The subject stood on a jump box of 45 cm height, behind the Opto-Jump. He was then instructed to step off towards the OptoJump optical measurement system in one motion and performed a maximal effort double-leg vertical jump, using an arm swing upon landing. The subject was instructed to quickly reverse the downward movement of the body to an upward movement in order to minimize the contact time with the ground upon landing, and maximize take-off velocity [20].

### Skating Multistage Aerobic Test (SMAT)

**FIG. 1:**
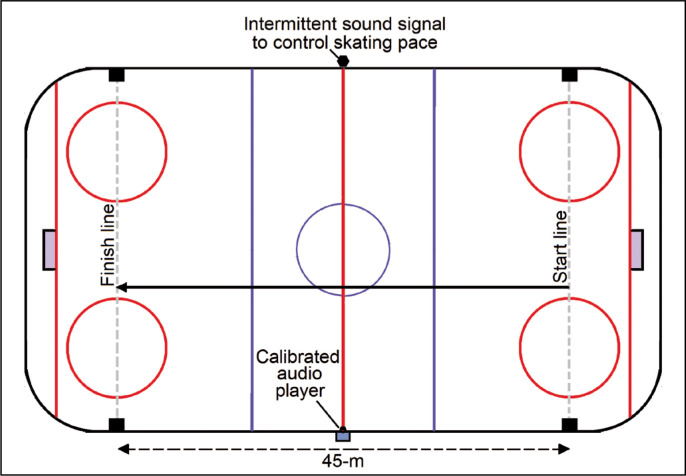
Diagram of Skating Multistage Aerobic Test (SMAT) layout [21].

This on-ice test for the prediction of VO_2max_ was designed by Leone et al. [21]. The test was conducted on a 45 m course defined with markers at both extremities of the indoor ice hockey rink (Figure 1). The participant skated, holding the hockey stick with the preferred single hand, from one end to the other with a predefined speed, and then the speed was gradually increased until the participant was unable to maintain the speed anymore (about 3 m before a marker after the sound signal). The pace was dictated by audible signals emitted by a calibrated audio player. The initial skating speed was set at 3.5 m · s^-1^, which was increased stepwise by 0.2 m · s^-1^. A 30 s rest period was allowed before beginning the next stage. The sound signals provided guidance to synchronize skating velocity with markers located at the midpoint (22.5 m) and both extremities of the 45 m course. At the start of each stage the participant player rested upright with the front skate parallel to the starting line. VO_2max_ was estimated from a table based on the formula [21]:


$${\text{VO}}_{2\text{max}}=22.368\times \left(\text{maximal velocity}\right)-58.618$$

### Forward and backward acceleration (0-5-10-30 m) test

**FIG. 2:**
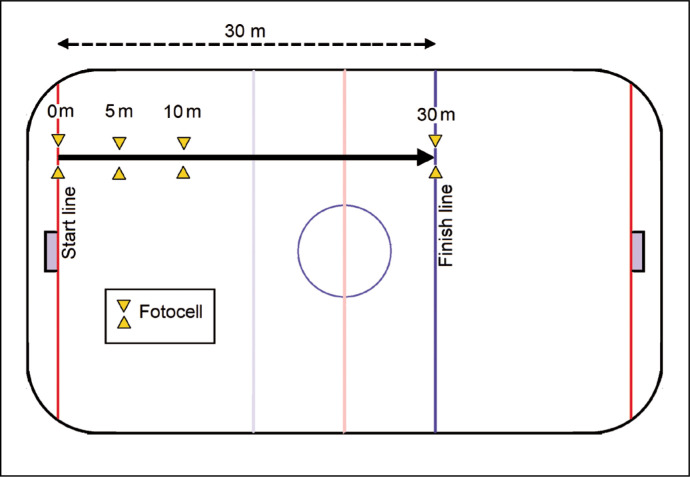
Setup for the forward and backward acceleration (0-5-10-30 m) test.

The subjects were required to start from the goal line and skate forward with maximal effort for 30 m distance on a straight line (Figure 2). All subjects performed this test twice, with a break of 10 min, and the fastest performance was recorded. During the break between two trials, players followed self-selected low intensity active recovery [13]. After a break of 15 min rest, backward skating was performed from the same starting line. The protocol of forward and backward skating was similar. Both the forward and backward skating started after the player remained at standstill close to the starting line. The forward and backward skating time and velocity were measured using a photocell automatic laser timing system (Microgate, Racetime 2, Bolzano, Italy). The starting and finishing sensors were placed 30 m apart on the ice. Sprint time was measured to the nearest 0.01 s. Photocells were set at 0, 5, 10, and 30 m distance. Average speed while skating forward was designated as forward average skating speed (FASS), and similarly, the average speed attained while skating backward was called backward average skating speed (BASS).

### Top speed test (50 ft flying top speed test)

**FIG. 3:**
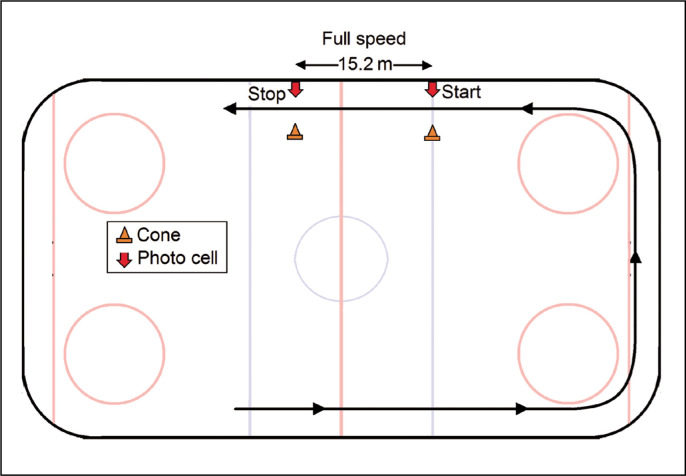
Setup for the top speed test.

Players were instructed to take their standing position on the goal line. The trial was started by skating toward the opposite goal line and around the net in an anticlockwise fashion toward the sprint line. An automatic laser timing system (Microgate, Racetime 2, Bolzano, Italy) measured the maximum speed while skating. The starting and finishing sensors were set on the opposing blue lines 50 ft apart (Figure 3). Time to calculate top speed was measured to the nearest 0.01 s. One trial was performed crossing over to the right and the other crossing over to the left. Rest time between sprints was 6 minutes, during which the player performed stretching and very light activities only [13].

### Repeated sprint ability (RSA) test [6 × (54 + 35) m]

**FIG. 4:**
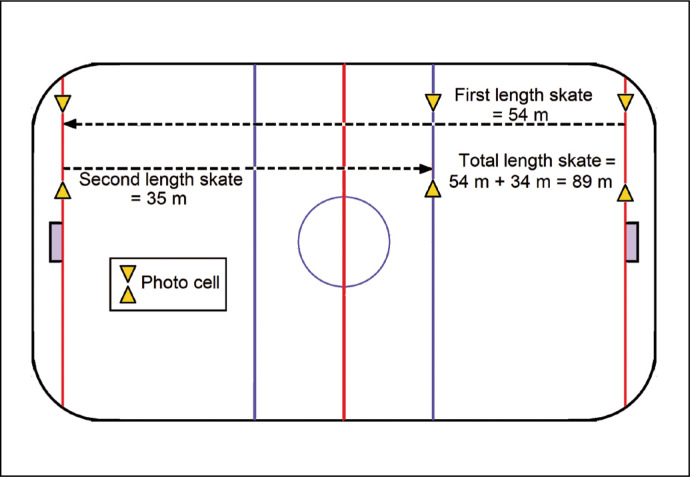
Schematic of the 6 x (54+35)-m repeated sprint ability (RSA) test.

This test involved sprinting an 89 m skating course – goal line to goal line (54 m) and back to the blue line (35 m) – as quickly as possible, and then returning to the starting line slowly. This was repeated 6 times, with 30 s recovery including the slow skating time required to return to the starting line. Figure 4 illustrates the course of skating of RSA. Subjects performed 5 minutes of individualized warm up before testing. All times were recorded by a set of photocells (Microgate Racetime 2, Bolzano, Italy). Fatigue index (FI) was calculated as follows [22]:


FI=(mean sprint time−fastest sprint time)/fastest time)×100(%)

### Statistical analyses

The descriptive statistics for all dependent variables were expressed by their means and standard deviations (mean ± SD). The reliability of all VJ tests were assessed using intraclass correlation analysis (ICC_2,1_) with 95% confidence intervals (± 95 CI). The coefficients obtained for CMJ, SJ, and DJ were 0.948 (0.794–0.981), 0.922 (0.809–0.967) and 0.913 (0.725–0.967), respectively. One-way analyses of variance were used to identify the statistical significance of the differences between vertical jumps. Tukey’s post-hoc tests were employed to determine the level of significance when a significant F-ratio was found. Homogeneity of variance was examined by conducting Levene’s test. The Pearson product–moment correlation coefficient was used to examine the relationship between off-ice and on-ice performance variables. The magnitude of the correlations was determined using the following criteria: trivial: 0 to 0.10, small: 0.11 to 0.3, moderate: 0.31 to 0.50, large: 0.51 to 0.70, very large: 0.71 to 0.90, and almost perfect: 0.91 to 1.00 [23]. Stepwise multiple regression was used to estimate on-ice performance variables by VJ (CMJ, SJ, DJ). All statistical analyses were conducted using Statistica 13.3 (TIBCO Software Inc., Palo Alto, CA, USA) and all graphics were plotted with the Sigma Plot version 12.0 and R software (R version 4.0.3, The R Foundation for Statistical Computing, Vienna, Austria). A level of p ≤ 0.05 was selected for statistical significance.

## RESULTS

Physical and physiological characteristics of the subjects, and the speeds they attained during on-ice speed tests (forward and backward acceleration test and top speed test), are presented in Table 1. VO_2max_ was estimated from SMAT as described. Both forward and backward skating speeds were highest in the 10 to 30 m distance range and lowest in the 0 to 5 m range. Forward average skating speed was 17% to 21% faster than BASS at different photocell distance markings.

**TABLE 1:** Physical and physiological characteristics of the subjects and skating speeds during on-ice speed tests.

Variables	Mean ± SD
**Physical characteristics**	
Age (y)	18.7 ± 2.2
Height (cm)	179.2 ± 11.6
Weight (kg)	77.8 ± 10.2
BMI (kg·m^-2^)	24.6 ± 1.9
VO_2max_ (ml·kg^-1^·mm^-1^)	69.5 ± 4.7

**Forward skating speed at distance zones**	
0–5 m (km·h^-1^)	18.91 ± 1.10
5–10 m (kim·h^-1^)	19.96 ± 0.91
10–30 m (km·h^-1^)	30.84 ± 1.13
0–30 m (km·h^-1^)	25.72 ± 0.92

**Backward skating speed at distance zones**	
0–5 m (km·h^-1^)	15.98 ± 1.52
5–10 m (km·h^-1^)	17.26 ± 1.42
10–30 m (km·h^-1^)	25.79 ± 1.43
0–30 m (km·h^-1^)	21.83 ± 1.30

**Maximum skating speed attained at 50-ft top skating speed test**	
Maximum speed (km·h^-1^)	35.10 ± 1.67
Duration at maximal speed (s)	1.54 ± 0.07

**FIG. 5:**
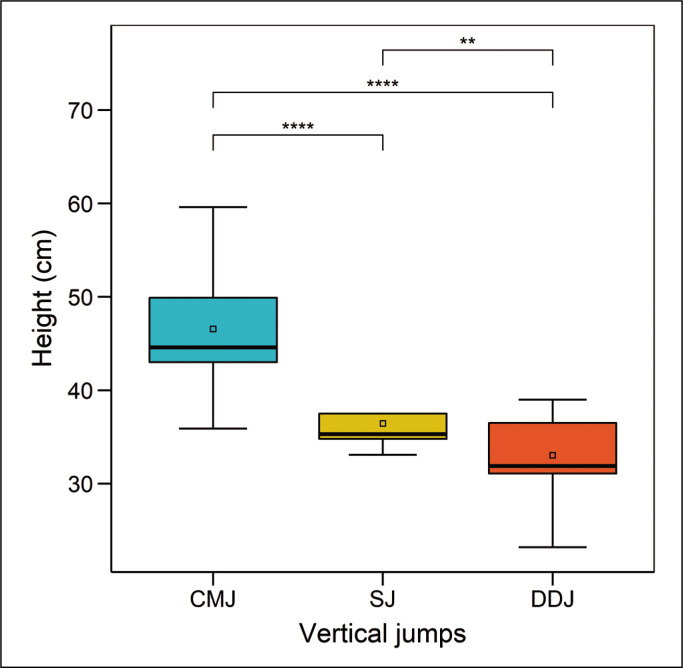
Jump test performance of the subjects. Note: *p<0.05, **p<0.01, ***p<0.001, ****p<0.0001.

Performance of all the jump tests, expressed in centimetres, is presented graphically in Figure 5. Countermovement jump score was the highest, followed by SJ and DDJ.

**FIG. 6:**
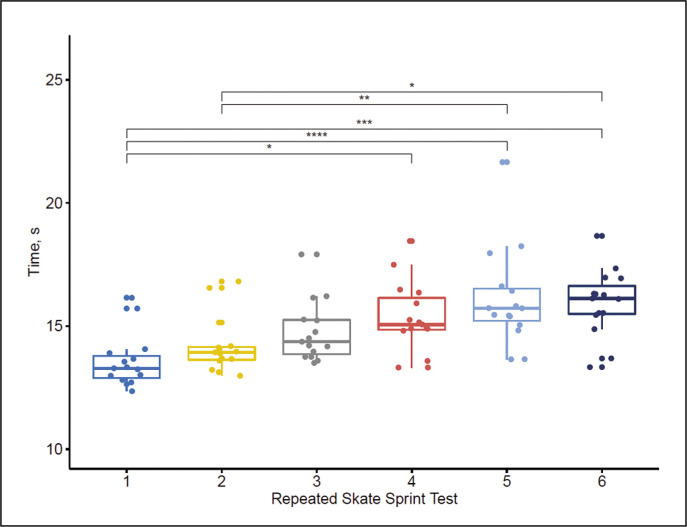
Duration of sprint bouts in repeated sprint ability test [6 x (54+35)-m] on ice. Note: *p<0.05, **p<0.01, ***p<0.001, ****p<0.0001.

Figure 6 shows the time required by the volunteers to complete each of the 6 skating sprints of the RSA test. The sprint time was shortest (13.19 ± 0.51 s) in the first bout, gradually increased thereafter, and was longest in the last (6^th^) bout (15.8 ± 1.97 s). The difference of the duration (and, thus speed) between any two consecutive bouts was non-significant. Statistical significance between sprint bouts and corresponding p-values are also presented in Figure 6.

**FIG. 7:**
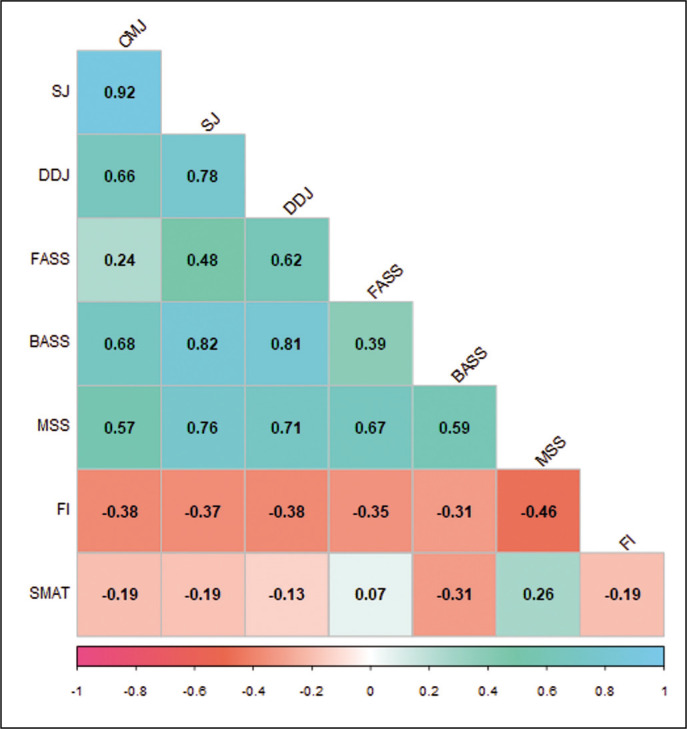
Correlations between various performance variables in ice hockey players.

Correlations between all the off-ice and on-ice performance of the subjects are presented in Figure 7. Countermovement jump shows a moderate correlation (r = 0.68, p < 0.05) with BASS but has no statistically significant correlation with any other ice skating fitness variables. Squat jump shows a strong correlation with both BASS (r = 0.82, p < 0.01) and MSS (r = 0.76, p < 0.01). Depth drop jump, on the other hand, shows moderate to strong correlations with all the skating speeds – FASS (r = 0.62, p < 0.05), BASS (r = 0.81, p < 0.01), and MSS (r = 0.71, p < 0.01). Performance decrement index (PDI) and fatigue index (FI), as calculated from RSA performance, are 11.07 ± 4.06 and 10.84 ± 3.47, respectively. Neither PDI nor FI shows a significant relationship with any of the off- or onice variables.

Correlations between all the VJ performances are significant; especially the correlation between CMJ and SJ is very strong (r = 0.92, p < 0.001). No valid relationship (r = 0.39) between FASS and BASS was found. SMAT (or predicted VO_2max_) was not found to be significantly correlated with any of the jump tests or with RSA, FASS, BASS, or MSS.

Based on the values obtained during VJ and on-ice tests, regression equations establishing the relationship between VJ and selected on-ice tests were determined by multiple regression analysis. Table 2 presents only those regression models for which the stochastic equation parameters are statistically significant. Regression analysis shows that CMJ and SJ are the best predictors for FASS over the first 5 m. Depth drop jump, on the other hand, predicts FASS over the distance of 30 m best. In the case of MSS, regression analysis reveals that SJ is an important predictor. Both DDJ and CMJ are the two valid indicators of BASS at the distance range of 10–30 m, whereas SJ can successfully predict backward skating performance (i.e., BASS) of the players over the distance of 30 m.

**TABLE 2:** Regression equations to predict on-ice performance from vertical jump tests.

Equations	R	R^2^	SEE	p-value
FASS 5 (km/h) = -0.291 CMJ (cm) + 0.513 SJ (cm) + 13.883	0.686	0.471	1.233	0.057
FASS 30 (km/h) = 0.128 DJ (cm) + 21.599	0.619	0.384	0.769	0.032
MSS (km/h) = 0.246 SJ (cm) + 25.965	0.760	0.577	1.106	0.004
BASS 10–30 (km/h) = 0.177 DJ (cm) + 0.047 CMJ (cm) + 17.600	0.889	0.790	0.640	0.009
BASS 30 (km/h) = 0.129 SJ (cm) + 16.527	0.825	0.680	0.474	0.006

Note: FASS, forward average skating speed; BASS, backward average skating speed; MSS, maximum skating speed; R, multiple correlation coefficient; R^2^, multiple R-Squared; SEE, standard error of estimate.

## DISCUSSION

The aim of this study was to quantify the association mainly between VJ and ice skating performance in junior ice hockey players. The key finding of this study is that the VJ performance is a good indicator of ice skating speed of junior ice hockey players although each of the three VJ studied has a different magnitude of correlation with skating performance. The DDJ is significantly correlated with all skating speeds – forward, backward, and maximum. Squat jump performance can predict BASS and MSS, whereas CMJ is only significantly correlated with BASS. Another important finding is that the RSA cannot be predicted either from any of the VJ tests or from aerobic fitness alone.

The major purpose of this study was to determine the specific vertical jump test(s) which is/are most appropriate to predict on-ice performance of junior ice hockey players. Strong explosive power in the legs is one of the prerequisites for success in ice hockey players [19, 24, 25]. This is required to skate fast in all possible directions, quick acceleration and deceleration, change of direction, blocking opponent players, and hitting the puck with the hockey stick. Unlike many other team sports, jumping is not a very common activity in the sport of ice hockey. However, various jump tests are commonly used to measure strength and explosive power of leg muscles. Vertical jump tests are probably the most widely used jump tests to evaluate the effectiveness and progression of training, and selection of talents [10].

A strong correlation (r = 0.92) between CMJ and SJ, as observed in this study, is highly expected because many of the primary muscles involved in both the jumps are common. However, their differences cannot be ignored either. Knee extensor muscles have been indicated as the primary muscles involved in SJ [26, 27], whereas ankle planter flexors, in addition to knee extensors, are considered as the primary muscles for CMJ [27, 28]. The lateral gastrocnemius muscle has been shown to be one of the strongest predictors of all the VJ – CMJ, SJ, and DDJ [29]. Depth drop jump performance can be influenced by the jump heights (e.g., 20, 40, 60 cm) [30].

In this study it was found that VJ, especially DDJ, was correlated with all the skating speed performances, but not with RSA. Vertical jump provides a reliable measure of strength and explosive power of lower limb muscles, especially muscles associated with ankle, knee, and hip joints [31, 32]. A number of anthropometric, biomechanical, and physiological factors regulate VJ performance [33, 34]. Joint angle, velocity of muscle contraction, centre of mass height, air resistance, acceleration, and momentum are some of the biomechanical factors regulating VJ. Powerful contraction of muscles such as extensors, gluteus maximus, quadriceps, hamstrings, gastrocnemius, and soleus push the body against gravity by ground reaction during VJ [35, 36]. Good muscle flexibility, especially in males, can positively influence VJ by accommodating the imposed stress more easily and efficiently [33]. The leading physiological determinants of VJ measurement are muscle fibre composition, phosphagen storage, and concentration of some specific enzymes in the muscles [35, 36].

The SJ and DDJ, in the present study, were moderately to strongly correlated with forward and backward average skating speed and maximal skating speed. Although these jumps are biomechanically different from ice skating, they involve many of the same leg muscles and share a similar underlying basis of power generation [2]. A number of other studies [3, 4] have shown a strong correlation between squat jump and skating speed. However, the results of Behm et al. [37] did not find any significant relationship between squat jump and skating speed, probably because of use of only two-thirds of the rink for the speed test, which was not enough to build up maximum speed. In their study, Behm et al. [37] did not find any relationship between drop-jump (a form of DDJ) and skating speed, because the period of contact with the ground was too short. Skating during playing ice hockey involves longer contact times, and unlike the drop jump, greater emphasis on impulse. Depth drop jump in this study was strongly correlated with FASS, BASS, and MSP. Longer contact time upon landing may be one of the reasons for such significant correlations.

All the vertical jumps in this study show a poor relationship with RSA time (nonsignificant in all cases). Vertical jumps in the laboratory are largely dependent on the explosive power and strong leg muscles, whereas FI, an RSA indicator, is strongly dependent on both anaerobic and oxidative systems of the ice hockey players. However, significant correlations between VJ and RSA performance have been found by some researchers [38, 39]. Countermovement jump was found to be significantly correlated with RSA by Buchheit et al. [40]. Similarly, a positive correlation between squat jump and 10-m shuttle run sprint performance was found by Wisløff et al. [39]. SMAT performance is mainly determined by the capabilities of the aerobic metabolism and not much influenced by the explosive power of the leg muscles or efficiency of anaerobic metabolism. This may explain why the SMAT results are not correlated with VJ or RSA test results.

The investigated on-ice and off-ice skills are only a few of many determinants of the complex performance of a hockey player. Onetime testing and reliability of the results are other limitations of the study. This study also did not focus on the playing position-specific experimental response of the players. This was due to time-limited organizational options of the research file. Although a number of studies, including EMG, revealed involvement of principal leg muscles in VJ, much of the information is still incomplete [27]. More specific laboratory and field studies are required that can reveal the role of leg muscles responsible for the remarkable correlation between off-ice and on-ice tests. Besides leg muscles, VJ requires coordinated activities of several other muscles in the trunk and arms. Future research should seek to reveal the role of these muscles in playing ice hockey, and appropriate laboratory and field tests that can predict on-ice performance of the players should be designed.

## Practical applications

The DDJ test, using a 45-cm high jump box, is the most reliable predictor of all three types of ice skating speeds – FASS, BASS, and MSS. Squat jump and CMJ tests can also predict some of the ice skating speeds. We recommend that CMJ, SJ, and DDJ tests can be used appropriately as off-ice tests on junior ice hockey players to predict their on-ice performance. The RSA test is commonly used to estimate anaerobic endurance of ice hockey players, but none of the VJ tests nor an endurance test like SMAT can be used to predict fatigue in ice hockey players. Backward average skating speed is about 18% slower than FASS, and this can help guide coaches to plan defence and attack phases of ice hockey matches. According to the findings of this study, the correlations between the test results may guide training plan design and follow-up in junior ice hockey players.

## Conflicts of Interest

The authors declare no conflict of interest.
